# The effectiveness, feasibility and acceptability of HIV service delivery at private pharmacies in sub‐Saharan Africa: a scoping review

**DOI:** 10.1002/jia2.26027

**Published:** 2022-10-26

**Authors:** Alexandra P. Kuo, Stephanie D. Roche, Melissa L. Mugambi, Jillian Pintye, Jared M. Baeten, Elizabeth Bukusi, Kenneth Ngure, Andy Stergachis, Katrina F. Ortblad

**Affiliations:** ^1^ Department of Pharmacy University of Washington Seattle Washington USA; ^2^ Public Health Sciences Division Fred Hutchinson Cancer Center Seattle Washington USA; ^3^ Department of Global Health University of Washington Seattle Washington USA; ^4^ School of Nursing University of Washington Seattle Washington USA; ^5^ Department of Epidemiology University of Washington Seattle Washington USA; ^6^ Department of Medicine University of Washington Seattle Washington USA; ^7^ Gilead Sciences Foster City California USA; ^8^ Centre for Microbiology Research Kenya Medical Research Institute Nairobi Kenya; ^9^ Department of Obstetrics and Gynecology University of Washington Seattle Washington USA; ^10^ Department of Community Health Jomo Kenyatta University of Agriculture and Technology Nairobi Kenya

**Keywords:** ART delivery, HIV testing, PrEP delivery, private pharmacies, scoping review, sub‐Saharan Africa

## Abstract

**Introduction:**

Private pharmacies are an understudied setting for differentiated delivery of HIV services that may address barriers to clinic‐delivered services, such as stigma and long wait times. To understand the potential for pharmacy‐delivered HIV services in sub‐Saharan Africa, we conducted a scoping review of the published and grey literature.

**Methods:**

Using a modified Cochrane approach, we searched electronic databases through March 2022 and HIV conference abstracts in the past 5 years for studies that: (1) focused on the delivery of HIV testing, antiretroviral therapy (ART) and/or pre‐exposure prophylaxis (PrEP) at private pharmacies in sub‐Saharan Africa; (2) reported on effectiveness outcomes (e.g. HIV incidence) or implementation outcomes, specifically feasibility and/or acceptability; and (3) were published in English. Two authors identified studies and extracted data on study setting, population, design, outcomes and findings by HIV service type.

**Results and discussion:**

Our search identified 1646 studies. After screening and review, we included 28 studies: seven on HIV testing, nine on ART delivery and 12 on PrEP delivery. Most studies (*n* = 16) were conducted in East Africa, primarily in Kenya. Only two studies evaluated effectiveness outcomes; the majority (*n* = 26) reported on feasibility and/or acceptability outcomes. The limited effectiveness data (*n* = 2 randomized trials) suggest that pharmacy‐delivered HIV services can increase demand and result in comparable clinical outcomes (e.g. viral load suppression) to standard‐of‐care clinic‐based models. Studies assessing implementation outcomes found actual and hypothetical models of pharmacy‐delivered HIV services to be largely feasible (e.g. high initiation and continuation) and acceptable (e.g. preferable to facility‐based models and high willingness to pay/provide) among stakeholders, providers and clients. Potential barriers to implementation included a lack of pharmacy provider training on HIV service delivery, costs to clients and providers, and perceived low quality of care.

**Conclusions:**

The current evidence suggests that pharmacy‐delivered HIV services may be feasible to implement and acceptable to clients and providers in parts of sub‐Saharan Africa. However, limited evidence outside East Africa exists, as does limited evidence on the effectiveness of and costs associated with pharmacy‐delivered HIV services. More research of this nature is needed to inform the scale‐up of this new differentiated service delivery model throughout the region.

## 1 INTRODUCTION

The delivery of HIV treatment and prevention services in high‐prevalence settings across sub‐Saharan Africa has largely been limited to public HIV clinics among certain other community settings [1, 2, 3]. In these settings, client‐level barriers to obtaining HIV services include lack of privacy, stigma associated with HIV clinics, and time spent travelling to and waiting at clinics. Clinic‐level barriers to delivering HIV services include understaffing and limited time with clients [4, 5]. To address these barriers, ministries of health and international multilateral agencies, such as the US President's Emergency Plan for AIDS Relief, are exploring differentiated service delivery (DSD) models [6] that vary who delivers services (e.g. nurses vs. clinicians [7]) as well as when and where services are being delivered (e.g. quarterly vs. biannual clinic visits [8], or family planning vs. HIV clinics [9]). During the COVID‐19 pandemic, the importance and development of these models increased to maintain access to essential HIV prevention and treatment services during periods of lockdown; for example, mobile clinics delivered HIV testing and pre‐exposure prophylaxis (PrEP) directly to communities [10, 11] and programmes for home delivery of HIV self‐tests [12] and antiretroviral therapy (ART) [13] were developed. As part of these DSD models, there is also growing interest in public–private partnerships that could enable new HIV service delivery models that meet client preferences and increase delivery efficiencies.

In recent years, DSD advocates have identified stand‐alone private pharmacies as a promising venue for the delivery of HIV services because they already provide sexual and preventive care services (e.g. contraception and treatment for sexually transmitted infections), point‐of‐care testing (e.g. for malaria and pregnancy) and monitoring of chronic conditions, such as diabetes [14, 15, 16, 17, 18, 19]. Private pharmacies also often outnumber public clinics in urban areas, provide a variety of services beyond healthcare (e.g. hygiene‐related products) and, as for‐profit entities, are financially motivated to prioritize client satisfaction (e.g. by maintaining client privacy around services sought). As such, for some clients seeking HIV services, private pharmacies may be better able to meet their desire for convenience, privacy and stigma‐free care than public clinics [20, 21, 22]. Additionally, adding HIV services to private pharmacies may be mutually beneficial to private pharmacies and public clinics by increasing business for the former and decreasing client volume for the latter [22].

We conducted a scoping review of the published and grey literature to identify emerging evidence on the effectiveness, feasibility and acceptability of private pharmacy‐delivered HIV services in sub‐Saharan Africa to highlight gaps in the literature and outline a future research agenda. We opted to conduct a scoping review rather than a systematic review because our primary objective was to identify the available evidence on this nascent HIV DSD model, not to appraise and synthesize evidence to inform practice and policy [23].

## 2 METHODS

### 2.1 Search strategy

To identify literature, we used a modified Cochrane approach [24] and followed the steps outlined in the Preferred Reporting Items for Systematic Reviews and Meta‐Analyses extension for Scoping Reviews checklist (Appendix S1) [25]. With the support of a research librarian at the University of Washington, we designed search terms that included “HIV,” “pharmacies,” “HIV service delivery” and “sub‐Saharan Africa” (for our full search terms, see Appendix S2). One author (APK) searched PubMed [26], Excerpta Medica dataBASE [27], Web of Science [28], and the Cumulative Index to Nursing and Allied Health Literature [29] to identify relevant literature published from inception to March 2022. We exported search results to Mendeley [30], a reference management software, and removed duplicate records.

To capture the grey literature (i.e. studies that have not yet been published in academic journals), we searched for pending or in‐press manuscripts and conference abstracts. Specifically, we searched registered randomized trials using ClinicalTrials.gov and the World Health Organization International Clinical Trials Registry Platform. To identify conference abstracts presented in the past 5 years (January 2017–March 2022), we searched the archives of the following international HIV conferences: the International AIDS Society Conference, the International AIDS Conference, the Conference on Retroviruses and Opportunistic Infections, and the HIV Research for Prevention Conference [31, 32, 33, 34, 35, 36]. Additionally, the co‐authors, many of whom are experts in HIV DSD models in sub‐Saharan Africa, helped identify grey literature not captured in the above‐described searches. Potentially relevant grey literature was added to Mendeley for review.

Prior to conducting our searches, we registered our methods to Open Science Framework (registration DOI: 10.17605/OSF.IO/HA7QK) [37].

### 2.2 Study selection

We included peer‐reviewed papers and conference abstracts that: (1) focused on the delivery of HIV services in private (not hospital‐based) pharmacies; (2) focused on the delivery of HIV testing, ART or PrEP; (3) were based in sub‐Saharan Africa; (4) reported on an effectiveness or implementation outcome (e.g. feasibility and/or acceptability); and (5) were published in English (Appendix S3). Studies measuring an effectiveness outcome were only included if they featured a control group for comparison. For studies measuring feasibility and/or acceptability, we recognized that the field currently lacks validated metrics for these outcomes and thus included studies where author‐defined outcome definitions were aligned with those commonly used in the HIV literature [38, 39]. Feasibility outcomes included costs associated with service delivery, healthcare personnel capacity, the ability to reach populations at increased risk of HIV acquisition and the uptake of services delivered. Acceptability outcomes included the willingness of clients/providers to uptake/deliver pharmacy‐based services, and client preferences for and barriers to uptake of pharmacy‐based services (e.g. stigma). Two authors (AK and KFO) applied the inclusion criteria by first screening the titles of identified studies, then the abstracts of selected titles. During the secondary screening, the same authors performed a full‐text review. Throughout, these authors noted the reasons for exclusion, and disagreements were resolved via discussion with co‐authors.

### 2.3 Data abstraction and synthesis

Author AK abstracted the following information from each included study: author; journal/conference; year of publication; country; population; study design (e.g. cross‐sectional survey, pilot study); outcome (effectiveness, feasibility and/or acceptability); the timing of outcome measurement (pre‐, mid‐ or post‐implementation); study objectives; and key findings on the outcome of interest. All authors reviewed the abstracted data and refined study findings by re‐reviewing manuscripts and conference abstracts. We summarized the findings by study and organized them by the HIV service delivered.

Quality assessment of included papers was completed by using the risk‐of‐bias tool (RoB2) for randomized trials assessing effectiveness outcomes and the risk‐of‐bias tool (ROBINS‐I) for non‐randomized studies assessing implementation outcomes. Quality assessments were summarized in a risk‐of‐bias assessment summary table using the risk‐of‐bias visualization (robvis) tool (Appendices S5–S8) [40].

## 3 RESULTS AND DISCUSSION

**Figure 1:**
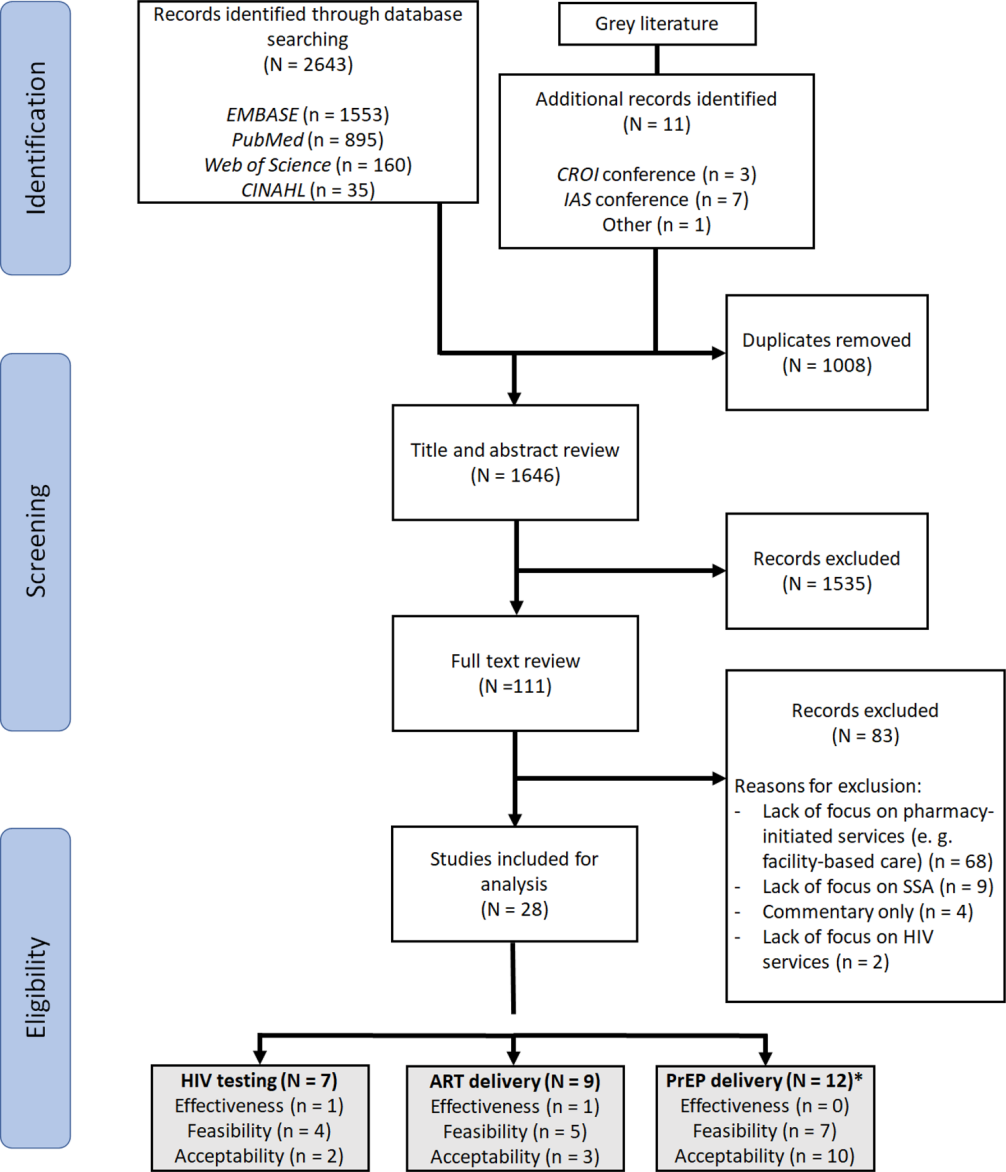
PRISMA diagram for the scoping review on differentiated models of community pharmacy‐delivered HIV services in sub‐Saharan Africa(SA). *The frequencies in this category sum to greater than the total number of studies because several studies were counted more than once (e.g. if the study was both a feasibility and acceptability study).

Our search identified 1646 studies: 1635 from the published literature and 11 from the grey literature (Figure 1). After the primary and secondary screening, we identified 28 studies for inclusion: seven on HIV testing, nine on ART delivery and 12 on PrEP delivery. Excluded studies primarily featured HIV service delivery in non‐private pharmacy settings and/or outside of sub‐Saharan Africa.

All included studies were published after January 2015, most (*n* = 18) between 2020 and 2021 (Appendix S4). Almost all studies were based in Southern (*n* = 9) or Eastern (*n* = 16) Africa, and about half (*n* = 12) were based in Kenya. Two studies measured effectiveness outcomes, while most (*n* = 26) measured an implementation outcome (*n* = 16 feasibility; *n* = 15 acceptability) at the pre‐ (*n* = 11), mid‐ (*n* = 2) or post‐intervention (*n* = 15) implementation stage. Study designs included randomized trials (*n* = 2), pilot studies (*n* = 11), cross‐sectional surveys (*n* = 4), retrospective analysis (*n* = 1), discrete choice experiments (*n* = 2), qualitative studies (*n* = 6) and stakeholder engagement (*n* = 2) (Table 1).

**Table 1:** **Descriptive characteristics of studies included in our review, *N* = 28** **^a^**

Characteristics	HIVST studies *N* = 7 (25%)	ART studies *N* = 9 (32%)	PrEP studies *N* = 12 (43%)^b^
UN region East Africa Middle Africa South Africa West Africa	5 (71%) 0 1 (14%) 1 (14%)	0 1 (11%) 5 (56%) 3 (33%)	11 (92%) 0 3 (25%) 0
Country income level Low and lower‐middle Upper‐middle	6 (86%) 1 (14%)	5 (56%) 4 (44%)	10 (83%) 3 (25%)
Study design Cross‐sectional survey Discrete choice experiment Pilot Qualitative Randomized trial^c^ Retrospective study Stakeholder meeting	1 (14%) 1 (14%) 4 (57%) 0 1 (14%) 0 0	1 (11%) 0 3 (33%) 3 (33%) 1 (11%) 1 (11%) 0	2 (17%) 1 (8%) 4 (33%) 3 (25%) 0 0 2 (17%)
Timing Pre‐implementation Mid‐implementation Post‐implementation	2 (29%) 0 5 (71%)	1 (11%) 0 8 (89%)	8 (67%) 2 (17%) 2 (17%)
Study population^b^ Adolescent girls and young women ART clients Males Pharmacy clients Pharmacy providers Stakeholders^d^ Young people	0 0 1 (14%) 6 (86%) 1 (14%) 7 (100%) 0	0 8 (89%) 0 0 1 (11%) 9 (100%) 0	2 (17%) 0 0 4 (33%) 2 (17%) 12 (100%) 3 (25%)
Primary outcome^b^ Acceptability Effectiveness Feasibility	2 (29%) 1 (14%) 4 (57%)	3 (33%) 1 (11%) 5 (56%)	7 (58%) 0 10 (83%)

Abbreviations: ART, antiretroviral treatment; HIVST, human immunodeficiency virus self‐test; PrEP, pre‐exposure prophylaxis; UN, United Nations; WHO, World Health Organization.

^a^ Data are shown for 28 included manuscripts and conference abstracts.

^b^ The frequencies and percentages in this category sum to greater than the total number of studies because several studies were counted more than once (e.g. if they included more than one study population).

^c^ The category “Randomized trial” includes both randomized control trials and cluster randomized trials.

^d^ The category “Stakeholders” includes groups of people who are identified by authors as stakeholders (such as policymakers) as well as pharmacy clients and providers.

In our quality assessment of the included literature (Appendices S5–S8), both of the randomized trials measuring effectiveness outcomes had one or more bias concerns. The majority (*n* = 23) of studies that measured implementation outcomes also had a “moderate” risk of bias, primarily due to selection bias.

### 3.1 HIV testing literature

We identified seven studies on pharmacy‐based HIV testing (Table 2). One effectiveness study, a randomized trial, explored the effect of HIV self‐testing (HIVST) vouchers redeemable at varying prices and distribution sites, including private pharmacies in urban areas, on service uptake [41]. Four studies explored the feasibility of [19, 42, 43, 44] and two studies explored the acceptability of [45, 46] of this HIV testing service delivery model. The studies measured all effectiveness and feasibility outcomes post‐implementation and acceptability outcomes pre‐implementation.

**Table 2:** Studies on models of community pharmacy‐based HIV testing delivery in sub‐Saharan Africa, *n* = 7

Publication	Countries	Population (size)	Study design (timing)	Study objective	Key findings
Effectiveness outcomes, *n* = 1					
Chang W et al., JAMA Network Open 2019 [41]	Zimbabwe	Pharmacy clients, ≥16 years (*n* = 3996)	Randomized trial *(post‐implementation)*	Examine how HIVST vouchers redeemable at varying prices and distribution sites, including pharmacies in urban areas, impacted the uptake of self‐test in urban and rural settings.	HIVST uptake was price sensitive, with higher uptake when free. ***The uptake of HIVST was higher with pharmacy versus clinic distribution in urban areas***; HIVST was 6.8% with pharmacy versus 2.9% with clinic distribution (aOR 2.8, 95% CI 1.7–4.5). Uptake was also higher at pharmacies among participants offered free vouchers.
Feasibility outcomes, *n* = 4					
Mugo P et al., Sex Transm Infect 2015 [19]	Kenya	Pharmacy clients, 18–29 years (*n* = 1490)	Pilot study *(post‐implementation)*	Determine the feasibility of pharmacy referral to clinic‐based HIV testing.	***Pharmacy referral to clinic‐based HIV testing was feasible***: quarter (24%) of targeted clients tested (especially clients seeking STI care).
Mugo P et al., PLOS One, 2017 [43]	Kenya	Pharmacy clients, ≥18 years (*n* = 463)	Pilot study *(post‐implementation)*	Assess the feasibility of pharmacy‐based HIV self‐test delivery.	***Pharmacy‐based HIV self‐test delivery is feasible and in high demand***; 35% of clients seeking services indicating HIV risk bought a self‐test; price ($1 USD) was not a barrier to uptake.
Kariuki T et al., IAS 2021^a^ [44]	Kenya	Pharmacy clients (*n* = 22,200)	Pilot study *(post‐implementation)*	Pilot test pharmacy distribution of HIVST versus HIV testing at health facilities.	From April to October 2020, 700 registered pharmacies sold 22,200 HIVST kits. ***Pharmacy‐delivered HIVST was feasible and reached more men than facility HIV testing***: at pharmacies, 69% of tests sold were to men >20 years compared to 38% at facilities.
Hacking D et al., AIDS Behav 2022 [42]	South Africa	Pharmacy and outreach event clients (*n* = 708)	Pilot study *(post‐implementation)*	Pilot test pharmacy distribution of HIVST and reported HIV testing outcomes.	***Pharmacy‐delivered HIV self‐test is feasible, but most who tested HIV positive already knew their HIV status***: 82% of clients reported any results from an HIV test; few reported testing HIV positive despite many reporting a previous HIV diagnosis.
Acceptability outcomes, *n* = 2					
Schaffer et al., Appl Health Econ Health Policy 2020 [45]	Uganda	Men, ≥18 years (*n* = 203)	Discrete choice experiment *(pre‐implementation)*	Elicit preferences and predict men's uptake of HIV testing using a discrete choice experiment.	Young men and men at elevated HIV risk had ***higher predicted uptake of HIVST with delivery at pharmacies*** versus other models of community delivery (e.g. home‐based testing).
Oseni and Erhun, Int. J. STD AIDS 2021 [46]	Nigeria	Pharmacy clients (*n* = 5840); community pharmacists (*n* = 71)	Cross‐sectional survey *(pre‐implementation)*	Assess the acceptability HTS at community pharmacies among providers and clients.	***Willingness to participate in HTS at community pharmacies was high among clients and providers***; 77% of clients indicated a willingness to participate and 91% of providers were willing to participate in training.

In general, these studies found that the delivery of HIV testing services at private pharmacies was effective, feasible and acceptable. The randomized trial found that in urban Zimbabwe, the uptake for HIVST was higher when at pharmacies than at clinics (6.8% vs. 2.9%, adjusted odds ratio 2.8, 95% confidence interval [CI] 1.7–4.5) [41]. The three pilot studies measuring feasibility found that, in Kenya, pharmacy referral to clinic‐based HIV testing and pharmacy delivery of HIVST was largely feasible, in high demand and reached key populations at HIV risk. Specifically, these studies found that a quarter of pharmacy clients (24%) referred to clinics for HIV testing followed through and received HIV testing (a service unavailable at private pharmacies in Kenya during the time of pilot implementation) [19]. Additionally, roughly a third of pharmacy clients (35%) seeking services indicating HIV risk purchased an HIV self‐test [43] and almost half (48%) of pharmacy HIV self‐tests sold were to clients >20 years old [44]. A pilot study in South Africa that found pharmacy‐delivered HIVST feasible also found that many clients who tested HIV positive already knew their HIV status [42]. The studies assessing acceptability, both qualitative, found that Ugandan men anticipated higher uptake of HIVST at a pharmacy versus other community delivery models [45] and that Nigerian pharmacy clients and providers were generally willing to engage in pharmacy‐delivered HIV testing services [46].

### 3.2 ART delivery literature

Of the nine studies on pharmacy‐based ART delivery (Table 3), one measured effectiveness outcomes, five measured feasibility outcomes and three measured acceptability outcomes. All outcomes, except one of the acceptability outcomes, were measured post‐implementation.

**Table 3:** Studies on models of community pharmacy‐based ART delivery in sub‐Saharan Africa, *n* = 9

Publication	Countries	Population (size)	Study design (timing)	Study objective	Key findings
Effectiveness outcomes, *n* = 1					
Fox MP et al., PLOS Med 2019 [47]	South Africa	ART clients (*n* = 578)	Cluster‐randomized trial *(post‐implementation)*	Assess the effect of differentiated medication delivery (DMD), including at private pharmacies, on HIV viral suppression.	***DMD patients had lower retention and comparable viral suppression to SOC patients***: viral suppression 77% with DMD and 74% with SOC (aRD –1.0%, 95% CI –12.2% to 10.1%).
Feasibility outcomes, *n* = 5					
Avong YK et al., PLOS One 2018 [48]	Nigeria	ART clients (*n* = 295)	Pilot study *(post‐implementation)*	Assess the feasibility of including pharmacies in models of differentiated ART delivery.	***Pharmacy ART refills for stable patients are feasible***: almost all (>99%) retained in care at retail pharmacies.
Ssuuna M et al., CROI 2020^a^ [49]	Uganda	ART clients (*n* = 9921)	Pilot study *(post‐implementation)*	Assess the feasibility of referring stable ART clients from public facilities to private pharmacies for refills.	***Using private pharmacies to delivery ART refills to stable clients feasible***: 30% clients accepted; 96% refilled ARTs as scheduled.
Pascoe SJS et al., J Int AIDS Soc. 2020 [50]	South Africa	Stakeholders (*n* = 88)	Qualitative study *(post‐implementation)*	Assess the feasibility of and barriers to differentiated medication delivery (DMD), including pharmacy delivery.	***Private pharmacists are an instrumental component of DMD, but have not traditionally been engaged with public sector care***; implementation challenges, such as medication supply and training, seen as a risk to adherence and retention.
Ibiloye OJ et al., IAS 2021^a^ [51]	Nigeria	ART clients (*n* = 210)	Pilot study *(post‐implementation)*	Assess the feasibility and clinical outcomes of devolving health facility clients to community pharmacies with multi‐month ART dispensing.	***Devolvement of patients into a pharmacy refill model was feasible without negatively impacting client outcomes***; all clients retained in care and almost all (99.5%) virally suppressed at 6 months.
Onovo A et al., CROI 2022^a^ [52]	Nigeria	ART clients (*n* = 85,245)	Retrospective analysis *(post‐implementation)*	Assess retention in decentralized drug distribution (DDD) ART delivery models, including a community pharmacy refill model, and examine factors associated with retention.	***The community pharmacy ART refills model had the highest median retention time compared to the other DDD models***: 73 months (95% CI 71–74) compared to <50 months in other models and was associated with long‐term ART retention.
Acceptability outcomes, *n* = 3					
Dorward J et al., BMJ Open 2020 [53]	South Africa	ART clients (*n* = 55) and healthcare workers (*n* = 8)	Qualitative study *(post‐implementation)*	Assess the acceptability of a centralized chronic medication dispensing distribution (CCMDD) programme, including ART, from community pick‐up points, including private pharmacies.	***ART refills at pharmacies as part of a CCMDD programme were acceptable with some limitations***; clients received quicker and more convenient services but some reported receiving inferior care compared to paying customers and concerns about inadvertently revealing their HIV status.
Mpofu M et al., JIAS 2021^a^ [54]	Botswana	ART clients (*n* = 61); private pharmacies (*n* = 42)	Cross‐sectional survey *(pre‐implementation)*	Assess the willingness to access/provider decentralizing ART refills through private pharmacies.	***Providing ART refills through private pharmacies was acceptable during COVID‐19***: 61% of clients willing to access refills there and pay for service, 100% of pharmacies willing to provide service.
Mukumbang FC et al., Qual Health Res. 2022 [55]	South Africa	ART clients (*n* = 68)	Qualitative study *(post‐implementation)*	Assess patients’ experiences with of three differentiated service delivery (DSD) ART models: facility adherence clubs, community adherence clubs and quick pharmacy pick‐up.	***Patients had mostly positive experiences with the quick pharmacy pick‐up model***: the model fit patients’ values, preferences and needs, but resulted in less information sharing, communication and education, and emotional/psychological support compared to the other DSD models.

Abbreviations: AGYWs, adolescent girls and young women; ARCs, adolescent refill clubs; aRD, adjusted risk difference; ART, antiretroviral therapy; CARC, community ART refill club; CCMDD, centralized chronic medication dispensing and distribution; CI, confidence interval; CPARP, community pharmacy ART refill programme; DMD, decentralized medication delivery; F‐CARGs, family‐centred ART refill groups; FGD, focus group discussion; IDI, in‐depth interview; ILWH, inmates living with HIV; PLHIV, people living with HIV; S‐CARG, self‐forming community ART refill groups; SRH, sexual and reproductive health services; VL, viral load.

^a^Conference abstract.

One randomized trial found that pharmacy‐delivered ART services could achieve similar patient‐level clinical outcomes compared to clinic‐delivered ART refill services [47]. In South Africa, stable ART clients who were randomized to getting refills at non‐clinic‐based pick‐up points, including private pharmacies, had comparable viral suppression (77.2%) to clients who continued to obtain refills at ART clinics (74.3%, adjusted risk difference [RD] –1.0%, 95% CI –12.2% to 10.1%), with a sub‐group of male participants in the intervention arm experiencing a non‐significant increase in viral suppression (RD 11.1%, 95% CI –3.4% to 25.5%) [47]. In this trial, the number of clients referred to private pharmacies over other pick‐up points was not specified and loss to follow‐up among ART clients in the intervention (16%, *n* = 24/275) and control (15%, *n* = 43/294) arms was similar.

The eight additional studies found that the delivery of ART services at private pharmacies was largely feasible and acceptable [48, 49, 50, 51, 52, 53, 54, 55]. Three pilot studies in Uganda and Nigeria that assessed the feasibility of pharmacy‐delivered ART refills found that the majority (>90%) of ART clients chose this option and refilled on schedule [49, 51, 56]. Additionally, another pilot study in Nigeria found that pharmacy‐delivered ART refills had the highest median retention time (73 months, 95% CI 71–74 months) compared to other differentiated drug distribution models (all <50 months) [52]. An analysis of in‐depth interviews with stakeholders in South Africa reported that private pharmacies were an instrumental and underexplored component of differentiated HIV service delivery [50]. In acceptability studies in South Africa and Botswana, ART clients reported that they would be willing to pay for pharmacy‐delivered ART refills [57], most found these services to be quicker and more convenient than clinic‐delivered refills [53], and many indicated that this model fit their values, preferences and needs [55]. However, some clients were concerned with inadvertent disclosure of their HIV status when pharmacy providers refill ART [53] and potentially lower‐quality communication, education and emotional support received with the delivery of ART refills at pharmacies versus clinics [55].

### 3.3 PrEP delivery literature

Of the 12 studies on pharmacy‐based PrEP delivery (Table 4), two measured feasibility outcomes, five measured acceptability outcomes and five measured both feasibility and acceptability outcomes. Most studies were based in Kenya (*n* = 9) and reported outcomes pre‐implementation (*n* = 8).

**Table 4:** Studies on models of community pharmacy‐based PrEP delivery in sub‐Saharan Africa, *n* = 12

Publication	Countries	Population (size)	Study design (timing)	Study objective	Key findings
Feasibility outcomes, *n* = 2					
Asewe, M et al., IAS 2021^a^ [58]	Kenya	Pharmacy clients (*n* = 527)	Pilot study *(mid‐implementation)*	Assess HIV risk behaviours among pharmacy clients as a barrier to PrEP delivery.	***The prevalence of behaviours associated with HIV risk was high among clients accessing SRH at retail pharmacies***: >60% reported partners with unknown HIV status or inconsistent condom use; 38% reported multiple sexual partners.
Ortblad KF et al., CROI 2022^a^ [60]	Kenya	Pharmacy clients (*n* = 287)	Pilot study *(post‐implementation)*	Assess the feasibility of initiation and continuation of PrEP at retail pharmacies.	***PrEP initiation and continuation at pharmacies is similar to or exceeds that at public clinics in Kenya***; 50% of clients screened initiated; continuation was 54% at 1 month, 35% at 4 months and 32% at 7 months.
Acceptability outcomes, *n* = 5					
Minnis AM et al., J Int AIDS Soc 2020 [67]	South Africa	Youth, 18–24 years (*n* = 807)	Discrete choice experiment *(pre‐implementation)*	Evaluate youth stated preferences for long‐acting PrEP attributes (with a focus on PrEP delivery).	Youth interested in long‐acting PrEP; ***young women preferred clinics versus pharmacy access; young MSM preferred pharmacy versus clinic access***.
Begnel ER et al., Int J STD AIDS 2020 [64]	Kenya	Community members, ≥18 to 34 years (*n* = 2617)	Cross‐sectional survey *(pre‐implementation)*	Assess community preferences and willingness to pay for pharmacy PrEP access.	***Pharmacy PrEP delivery of interest to community***: 38% reported as preferred venue and 61% of these people willing to pay.
Dietrich JJ et al., BMC Health Serv Res. 2021 [65]	South Africa	Young people, ≥13 to 24 years (*n* = 74)	Qualitative study *(pre‐implementation)*	Measure young peoples’ preferences for daily and on‐demand PrEP to identify critical attributes.	***Young people preferred pharmacy‐ versus clinic‐based PrEP delivery***; compared to clinic‐based PrEP delivery, pharmacy‐based delivered was perceived to be more private, confidential and faster.
Nakambale HN et al., IAS, 2021 [61]^a^	Kenya	Pharmacy clients (*N* = 253)	Pilot study *(mid‐implementation)*	Measure client willingness to pay for pharmacy‐based PrEP.	***Almost all participants (96%) were willing to pay for pharmacy‐delivered PrEP services*** and many (31%) were willing to pay more than they were charged (>300 KSH) for these services.
Tubert J. et al., AIDS Res Ther. 2021 [66]	Tanzania	AGYW (*n* = 56) and drug shops (*n* = 26)	Cross‐sectional survey *(pre‐implementation)*	Assess community preferences in supplying PrEP (oral pills and the dapivirine ring) among drug shops and AGYW.	***PrEP delivery (oral pills and dapivirine ring) to AGYW at community drug shops is acceptable***: almost all (85%) providers said they would provide PrEP and many (oral: 64%; ring: 43%) AGYW clients said they would be interested in PrEP access here.
Feasibility and acceptability outcomes, *n* = 5					
USAID/PEPFAR, ICASA 2017^a^ [6]	Kenya, South Africa and Zimbabwe	Stakeholders (*n* = 30)	Review + stakeholder engagement *(pre‐implementation)*	Analyse the private sector's ability to support the rollout of oral PrEP to women.	***Pharmacies have a “medium” opportunity to delivery PrEP***: have high reach/acceptability, but lack trained HCWs with prescribing capacity.
Ortblad KF et al., BMC Health Serv Res. 2020 [62]	Kenya	PrEP stakeholders (*n* = 36)	Stakeholder meeting *(pre‐implementation)*	Collaboratively develop a care pathway for pharmacy‐based PrEP delivery via stakeholder meeting.	***PrEP delivery stakeholders were strongly supportive of pharmacy PrEP***: developed care pathway for pilot testing in Kenya.
Roche S et al., AIDS Behav. 2021 [63]	Kenya	Pharmacy clients (*n* = 40) and providers (*n* = 16)	Qualitative study *(pre‐implementation)*	Assess determinants of the acceptability/feasibility of pharm. PrEP among providers and clients.	***Pharmacy PrEP delivery is perceived to be feasible and acceptable among providers and clients*** if PrEP care is private, respectful, safe and affordable.
Wairimu N et al., HIVR4P 2021^a^ [69]	Kenya	Clinic (*N* = 10) and pharmacy (*N* = 16) providers	Qualitative study *(pre‐implementation)*	Assess PrEP and pharmacy providers willingness to collaborate for PrEP delivery.	***PrEP and pharmacy providers are ready and willing to work together*** in public–private partnerships for PrEP delivery.
Pintye J et al., IAS 2021^a^ [59]	Kenya	AGYW, ≥15 to 24 years (*n* = 208)	Pilot study *(post‐implementation)*	Pilot a nurse‐initiated model of PrEP delivery at retail pharmacies targeting AGYWs.	***Nurse‐initiated pharmacy PrEP uptake was high among AGYW accessing SRH services and most were willing to pay for this service***; update was 86% among those offered PrEP and 63% reported willingness to pay; PrEP uptake among AGYWs at pharmacies much higher than at family planning clinics.

Abbreviations: AGYWs, adolescent girls and young women; MSM, men who have sex with men; PrEP, pre‐exposure prophylaxis; SRH, sexual and reproductive health services; VL, viral load.

^a^Conference abstract/grey literature.

The studies largely found that pharmacy‐delivered PrEP was feasible and acceptable or anticipated under certain criteria. Two pilot studies in Kenya, one that had pharmacy providers deliver PrEP (hereafter, “pharmacy provider‐led model”) and another that stationed nurses in pharmacies to deliver PrEP (hereafter, “nurse‐led model”), effectively reached populations with HIV risk [58], had similar or higher levels of PrEP initiation and continuation compared to clinic‐based models [59, 60] and found that clients were willing to pay for the pharmacy‐delivered PrEP [59, 61]. Prior to implementing these and other pilots, stakeholders in Kenya, South Africa and Zimbabwe reported perceiving private pharmacies as having a “medium” opportunity to deliver PrEP so long as pharmacy providers received proper training [6]. Policymakers and representatives from professional bodies and civil society in Kenya collaboratively developed the pharmacy provider‐led model [62]. In formative research, Kenyan pharmacy providers and clients anticipated pharmacy‐delivered PrEP services would be feasible and acceptable as long as services were private, respectful, safe and affordable [63]. PrEP clinicians and pharmacy providers expressed willingness to collaborate to deliver PrEP in pharmacies. Other studies found that pharmacy‐delivered PrEP was of interest to community members in Kenya [64], preferable to clinic‐based delivery among young people in South Africa [65] and of interest to adolescent girls and young women (AGYW) in Tanzania [66]. However, one study in South Africa found that young women anticipated they would prefer clinics to pharmacies for accessing long‐acting PrEP once it becomes available [67].

## 4 DISCUSSION

Early evidence on private pharmacy‐based HIV service delivery suggests that in most sub‐Saharan African settings studied, these models increase service uptake without jeopardizing clinical outcomes and are largely feasible and acceptable among clients, providers and other key stakeholders. The effectiveness evidence on this HIV service delivery model, however, is quite limited—we only identified two randomized trials [41, 47]—demonstrating the need for more data of this nature to inform the scale‐up of this HIV service delivery model. In many studies, pharmacy clients reported interest in, or preference for, pharmacy‐delivered HIV testing, ART refills and PrEP [43, 48, 66, 68], and engaged in these services when given the opportunity [58, 60]. Stakeholders from diverse organizations, including professional bodies and regulatory agencies, perceived private pharmacies as an instrumental component of differentiated HIV service delivery [6, 50] and were willing to help collaboratively design and implement models of PrEP service delivery in this setting [62, 69]. Additionally, many clients reported a willingness to pay for [59, 61] and use these services for increased convenience, autonomy and privacy [63]. Some limitations of pharmacy‐delivered HIV services included a lack of training and prescribing privileges among pharmacy providers [6, 50, 62] and concerns about HIV status disclosure and quality of care among pharmacy clients [53, 55].

In the studies that measured feasibility outcomes, the review found that pharmacy‐delivered HIV services can reach those in need who may not otherwise engage in traditional clinic‐based services. For example, several studies indicated that clients who routinely accessed private pharmacies for sexual and reproductive health services, such as emergency contraception and sexual performance‐enhancing drugs, frequently reported behaviours associated with the risk of HIV acquisition (e.g. multiple sexual partners) [43, 45, 58, 59, 66, 70]. In the pilot study of the nurse‐led PrEP delivery model in Kenya, HIV risk behaviours, such as engaging in transactional sex or sex with a partner of unknown HIV status, were significantly more prevalent among AGYW accessing sexual and reproductive health services at private pharmacies than at public family planning clinics [59]. Additionally, in the pharmacy provider‐led PrEP pilot in Kenya, over half of the clients initiating PrEP were men [60]. This evidence suggests that pharmacy‐delivered HIV services could be complementary to, rather than duplicative of, services delivered at public clinics and could potentially help expand the reach of services to populations not currently engaged in traditional care models. Recent randomized trials have demonstrated that even with very high levels of population‐level HIV treatment coverage, HIV incidence persists [71]; thus emphasizing the need for additional DSD models, like pharmacy‐based PrEP delivery.

Additionally, in the studies that measured feasibility outcomes, client HIV service uptake and continuation at private pharmacies was comparable to, or higher than, that at public clinics [41, 46, 47, 48, 49, 51, 52, 55, 72]. This could be attributable to the increased convenience and efficiency of pharmacy‐delivered services; private pharmacies are more prevalent in many sub‐Saharan African settings, especially in urban areas, and generally deliver faster services than public clinics [65]. However, we cannot rule out the possibility that these results are due, in part or in whole, to selection bias and/or to the lack of alternative service delivery models in some studies. For example, in many of the pharmacy‐delivered ART refill models, only stable ART clients were given the option to refill at pharmacies, and in some models, clients were not given the option to choose a clinic‐based refill model [54]. Therefore, additional research among clients who struggle to engage in clinic‐based HIV services is needed to understand whether pharmacy‐based models are a good alternative. In such research, participants should be given a choice and allowed to select the model of delivery that best fits their needs and preferences [60, 73].

In the studies that measured acceptability outcomes, our review suggests that while pharmacy‐delivered HIV services are perceived to be largely acceptable, this may be conditional on pharmacy providers’ ability to maintain clients’ privacy [46, 57, 63, 65, 66] and provide high‐quality services [53, 55, 63, 67]. As such, implementers should consider interventions that might increase the privacy of pharmacy‐delivered HIV services, such as integrating self‐screening tools for HIV risk [74, 75], delivering antiretroviral drugs in discrete packaging [76, 77] and counselling clients in a private setting [62]. Additionally, this finding emphasizes the need for proper training of pharmacy providers on HIV service delivery to help ensure the quality and acceptability of services provided, prevent the spread of misinformation and reduce risks of substandard client counselling, which could lead to antiretroviral misuse and drug resistance [50, 78].

Many of the studies that measured acceptability outcomes also suggested a link between the acceptability of pharmacy‐delivered HIV services and service affordability [41, 43, 57, 61]. Determining the cost of these services to clients, pharmacies and third‐party payers, like ministries of health, will be critical. The studies included in our review suggest that people living with or at risk of acquiring HIV are willing to pay for pharmacy‐delivered HIV services [43, 59, 61, 63]. Potential advantages of charging clients for pharmacy‐delivered HIV services include increased perceived service quality and use by clients [79], a greater incentive for pharmacies to deliver services and long‐term sustainability of these models, particularly in cases of decreasing donor support. More costing research, especially modeling studies, is needed to better understand how cost‐sharing models might support public–private partnerships that could facilitate the sustainable scale‐up of HIV services at private pharmacies in sub‐Saharan Africa [69].

Although this review suggests that pharmacy‐delivered HIV services may be effective, feasible and acceptable, additional research is needed to identify potential ways to overcome barriers to scale‐up. First, regulatory pathways for pharmacy‐delivered HIV services must be established. Pharmacy providers in sub‐Saharan Africa (most of whom are pharmaceutical technologists or diploma pharmacists) are not legally allowed to prescribe antiretrovirals, and many are not allowed to administer HIV testing, unless offered through pilot project approval or certification provisions. However, guidelines and regulations on this are rapidly changing. In Kenya, the Ministry of Health has granted pharmacy providers engaged in select research studies special permission to prescribe PrEP using a checklist and remote clinician oversight [62]. In South Africa, a certification known as the “pharmacist‐initiated management of antiretroviral therapy” (PIMART) is currently under review with the South African Department of Health. If approved, PIMART‐certified pharmacists will be allowed to prescribe and manage the delivery of antiretrovirals, including PrEP, ART and post‐exposure prophylaxis [78]. Second, national electronic medical record (EMR) systems for tracking government‐ or donor‐issued HIV drugs are not currently in use in sub‐Saharan Africa outside of clinical settings. Understanding how these systems might be adapted to support drug delivery in community settings while maintaining integration with the national EMR system and accurate client linkage through the use of a unique identification code is important not just for the success of pharmacy‐delivered models but also for other community‐delivered models, such as peer and door‐to‐door delivery. Finally, the potentially high costs of these services to pharmacy clients, particularly in the absence of government or donor subsidies, present a potential access barrier that merits further investigation.

This review had several limitations. First, this review was focused on HIV service delivery at private pharmacies in sub‐Saharan Africa and did not focus on other types of community delivery, thus limiting the scope. Second, our review was limited to published academic papers and conference abstracts, and thus did not include reports, briefs and write‐ups from related implementation projects. Third, the models of pharmacy‐delivered HIV services captured in this review were not uniform, thus precluding direct comparison of study outcomes. Fourth, feasibility and acceptability are multifaceted concepts for which the field of implementation research has yet to establish standard, validated metrics. Fifth, the majority of studies were conducted in Kenya, one of several lower‐middle‐income countries in sub‐Saharan Africa, thus limiting the generalizability of our findings to other countries in this region. Finally, in some of the included studies, not all participants were given the choice of an alternative, non‐pharmacy‐based HIV service delivery model, which may have biased the outcomes.

## 5 CONCLUSIONS

Private pharmacies have the potential to play an important role in the differentiated delivery of HIV services in sub‐Saharan Africa and in ending the AIDS epidemic by 2030 [6]. Pharmacies may help reach populations whose HIV prevention and treatment needs are not being met with traditional models of clinic‐based service delivery. Emerging evidence suggests that pharmacy‐delivered HIV services may be acceptable and feasible in diverse populations; however, few studies have measured the effectiveness of these models [41, 47]. To better understand the generalizability of these findings, more research is needed in other countries beyond Kenya and South Africa that have different political and economic environments. As some countries in sub‐Saharan Africa move forward with testing models of pharmacy‐delivered HIV services, there is a pressing need to identify barriers to implementation and test strategies that may optimize the reach and sustainability of these models. Additionally, more randomized trials are needed to determine the effectiveness of pharmacy‐delivered HIV services compared to pharmacy referral to clinic‐delivered HIV services, which is the limit of most pharmacy providers’ scope of practice at the moment. For these models to be sustainable over time, frameworks for public–private partnerships will have to be established and EMR systems that can accurately track the distribution of public commodities in private settings and integrate with established systems will need to be developed. With the regulatory approval for long‐acting forms of antiretrovirals on the horizon in sub‐Saharan Africa [80, 81] and the COVID‐19 epidemic underscoring the need to decongest crowded public clinics [82, 83], the time to develop, test and scale‐up pharmacy‐delivered HIV services is now.

## COMPETING INTERESTS

JMB has received donations of study medication from Gilead Sciences and serves on advisory committees for Gilead Sciences, Merck and Janssen. For the remaining authors, none were declared.

## AUTHORS’ CONTRIBUTIONS

APK, SDR, AS and KFO designed this scoping review. APK and KFO analysed the data and wrote the first draft of this manuscript. All authors edited the draft, provided insights and approved the final manuscript for publication.

## FUNDING

This work was supported, in whole or in part, by the Bill & Melinda Gates Foundation [INV‐029935, PI: Ortblad]. Under the grant conditions of the Foundation, a Creative Commons Attribution 4.0 Generic License has already been assigned to the Author Accepted Manuscript version that might arise from this submission. In addition, this review was supported by the National Institute of Mental Health (R34 MH120106, PI: Ortblad; K99 MH121166, PI: Ortblad), and the National Institute of Allergy and Infectious Disease (P30 AI027757, PI: Celum). Since this was a scoping review, no primary data were collected for analysis. All presented data are published and available in the public domain or upon request.

## Supporting information


**Appendix S1**. PRISMA‐ScR Checklist.
**Appendix S2**. Search teams for the scoping review on models of pharmacy‐delivered HIV services in sub‐Saharan Africa.
**Appendix S3**. PICOS criteria for study inclusion in the scoping review.
**Appendix S4**. Studies identified on differentiated models of community pharmacy‐based HIV service delivery in SSA, by publication year.
**Appendix S5**. Traffic light plot ‐ quality assessment of extracted literature using ROBINS‐I.
**Appendix S6**. Summary plot ‐ quality assessment of extracted literature using ROBINS‐I.
**Appendix S7**. Traffic light plot ‐ quality assessment of extracted literature using ROB2.
**Appendix S8**. Summary plot ‐ quality assessment of extracted literature using ROB2.

## Data Availability

Data sharing is not applicable to this article as it is a review and no new data were generated during the current study.
