# Supplementary material for: The effectiveness, feasibility and acceptability of HIV service delivery at private pharmacies in sub‐Saharan Africa: a scoping review
Source: J Int AIDS Soc. 2022 Oct 26;25(10):e26027. doi: 10.1002/jia2.26027 (PMC9597376; doi:10.1002/jia2.26027)

**Appendix 1. PRISMA-ScR Checklist**

| **Section** | **Item #** | **PRISMA-ScR Checklist Item Description** | **Line Reported** |
| --- | --- | --- | --- |
| **Title** | | | |
|  | 1 | Identify the report as a scoping review | 96 |
| **Abstract** | | | |
| Structured summary | 2 | Provide a structured summary that includes (as applicable): background, objectives, eligibility criteria, sources of evidence, charting methods, results, and conclusions that relate to the review questions and objectives. | 96-101 |
| **Introduction** | | | |
| Rationale | 3 | Describe the rationale for the review in the context of what is already known. Explain why the review questions/objectives lend themselves to a scoping review approach. | 99-101 |
| Objectives | 4 | Provide an explicit statement of the questions and objectives being addressed with reference to their key elements (e.g., population or participants, concepts, and context) or other relevant key elements used to conceptualize the review questions and/or objectives | 96-98 |
| **Methods** | | | |
| Protocol and registration | 5 | Indicate whether a review protocol exists; state if and where it can be accessed (e.g., a Web address); and if available, provide registration information, including the registration number | 110-111 |
| Eligibility criteria | 6 | Specify characteristics of the sources of evidence used as eligibility criteria (e.g., years considered, language, and publication status), and provide a rationale. | 87-107 |
| Information sources | 7 | Describe all information sources in the search (e.g., databases with dates of coverage and contact with authors to identify additional sources), as well as the date the most recent search was executed. | 91-109 |
| Search | 8 | Present the full electronic search strategy for at least 1 database, including any limits used, such that it could be repeated. | 93-98, Appendix 2 |
| Selection of sources of evidence | 9 | State the process for selecting sources of evidence (i.e., screening and eligibility) included in the scoping review. | 113-125 |
| Data charting process | 10 | Describe the methods of charting data from the included sources of evidence (e.g., calibrated forms or forms that have been tested by the team before their use, and whether data charting was done independently or in duplicate) and any processes for obtaining and confirming data from investigators. | 125-129 |
| Data items | 11 | List and define all variables for which data were sought and any assumptions and simplifications made. | 131-136 |
| Critical appraisal of individual sources of evidence | 12 | If done, provide a rationale for conducting a critical appraisal of included sources of evidence; describe the methods used and how this information was used in any data synthesis (if appropriate).1 | 138-142 |
| Synthesis of results | 13 | Describe the methods of handling and summarizing the data that were charted. | 149-156 |
| **Results** | | | |
| Selection of sources of evidence | 14 | Give numbers of sources of evidence screened, assessed for eligibility, and included in the review, with reasons for exclusions at each stage, ideally using a flow diagram. | 144-146, Figure 1 |
| Characteristics of sources of evidence | 15 | For each source of evidence, present characteristics for which data were charted and provide the citations. | 149-156, Table 1 |
| Critical appraisal within sources of evidence | 16 | If done, present data on critical appraisal of included sources of evidence (see item 12). | 157-160, Appendix 5-8 |
| Results of individual sources of evidence | 17 | For each included source of evidence, present the relevant data that were charted that relate to the review questions and objectives. | 157-236 |
| Synthesis of results | 18 | Summarize and/or present the charting results as they relate to the review questions and objectives. | 218-236 |
| **Discussion** | | | |
| Summary of evidence | 19 | Summarize the main results (including an overview of concepts, themes, and types of evidence available), link to the review questions and objectives, and consider the relevance to key groups | 238-325 |
| Limitations | 20 | Discuss the limitations of the scoping review process | 326-337 |
| Conclusions | 21 | Provide a general interpretation of the results with respect to the review questions and objectives, as well as potential implications and/or next steps | 305-325 |
| **Funding** | | | |
| Funding | 22 | Describe sources of funding for the included sources of evidence, as well as sources of funding for the scoping review. Describe the role of the funders of the scoping review. | 368-377 |

| **Appendix 2. Search teams for the scoping review on models of pharmacy-delivered HIV services in sub-Saharan Africa**  ^1^Countries were selected based off of sub-Saharan African classification according to the United Nations Development Programme https://www.africa.undp.org/content/rba/en/home/regioninfo.html |
| --- |
| **Terms to identify differentiated models of pharmacy-based HIV testing** |
| (“HIV testing” OR “HIV test” OR “HIVST” OR “HIV self test” OR “HIV self-test” OR “human immunodeficiency virus test” OR “human immuno-deficiency virus test” OR “human immunedeficiency virus test” OR “human immune deficiency virus test” OR “HIV screening” OR “HIV screen” OR “HIV diagnos*” OR “HIV serodiagnos*”) AND (“HIV” OR “human immunodeficiency virus” OR “human immuno-deficiency virus” OR “human immunedeficiency virus” OR “human immune deficiency virus”) AND (“pharmacy” OR “pharmacies” OR “pharmac” OR “pharmacy access” OR “pharmacy initiated” OR “pharmacy-based” OR “community pharmacy services” OR “chemist” OR “drug shop” OR “external pick-up-point” OR “external pick up point” OR “Ex-PuPs” OR “Ex PuPs” OR “decentralized medication delivery” OR “DMD” OR “retail pharmac” OR “community pharmac”) AND (“sub-Saharan Africa” OR “subsaharan Africa” OR “SSA” OR “Angola” OR “Benin” OR “Botswana” OR “Burkina faso” OR “Burundi” OR “Cameroon” OR “Cape Verde” OR “Central African Republic” OR “Chad” OR “Comoros” OR “DRC” OR “Democratic Republic of Congo” OR “Republic of Congo” OR “Cote divoire” OR “Cote d Ivoire” OR “Equatorial Guinea” OR “Eritrea” OR “Ethiopia” OR “Gabon” OR “Gambia” OR “Ghana” OR “Guinea” OR “Guinea-Bissau” OR “Kenya” OR “Lesotho” OR “Liberia” OR “Madagascar” OR “Malawi” OR “Mali” OR “Mauritania” OR “Mauritius” OR “Mozambique” OR “Nambia” OR “Niger” OR “Nigeria” OR “Rwanda” OR “Sao Tome and Principe” OR “Sao Tome & Principe” OR “Senegal” OR “Seychelles” OR “Sierra Leone” OR “South Africa” OR “South Sudan” OR “Sudan” OR “Swaziland” OR “Tanzania” OR “Togo” OR “Uganda” OR “Zambia” OR “Zimbabwe”) |
| **Terms to identify differentiated models of pharmacy-based ART delivery^1^** |
| (“Antiretroviral” OR “Antiretroviral delivery” OR “anti-retroviral delivery” OR “ARV delivery” OR “ART” OR “antiretroviral therapy” OR “anti-retroviral therapy”) AND (“HIV” OR “human immunodeficiency virus” OR “human immuno-deficiency virus” OR “human immunedeficiency virus” OR “human immune deficiency virus”) AND (“Nonprescription Drugs” OR “nonprescription” OR “over the counter” OR “without a prescription” OR “pharmacist-prescribed” OR “pharmacy” OR “pharmacies” OR “pharmac” OR “pharmacy access” OR “pharmacy initiated” OR “pharmacy-based” OR “community pharmacy services” OR “chemist” OR “drug shop” OR “external pick-up-point” OR “external pick up point” OR “Ex-PuPs” OR “Ex PuPs” OR “decentralized medication delivery” OR “DMD” OR “retail pharmac” OR “community pharmac”) AND (“sub-Saharan Africa” OR “subsaharan Africa” OR “SSA” OR “Angola” OR “Benin” OR “Botswana” OR “Burkina faso” OR “Burundi” OR “Cameroon” OR “Cape Verde” OR “Central African Republic” OR “Chad” OR “Comoros” OR “DRC” OR “Democratic Republic of Congo” OR “Republic of Congo” OR “Cote divoire” OR “Cote d Ivoire” OR “Equatorial Guinea” OR “Eritrea” OR “Ethiopia” OR “Gabon” OR “Gambia” OR “Ghana” OR “Guinea” OR “Guinea-Bissau” OR “Kenya” OR “Lesotho” OR “Liberia” OR “Madagascar” OR “Malawi” OR “Mali” OR “Mauritania” OR “Mauritius” OR “Mozambique” OR “Nambia” OR “Niger” OR “Nigeria” OR “Rwanda” OR “Sao Tome and Principe” OR “Sao Tome & Principe” OR “Senegal” OR “Seychelles” OR “Sierra Leone” OR “South Africa” OR “South Sudan” OR “Sudan” OR “Swaziland” OR “Tanzania” OR “Togo” OR “Uganda” OR “Zambia” OR “Zimbabwe”) |
| **Terms to identify differentiated models of pharmacy-based PrEP delivery** |
| (“Pre-exposure prophylaxis” OR “preexposure prophylaxis” OR “antiretroviral prophylaxis” OR “preexposure chemoprophylaxis” OR “pre-exposure chemoprophylaxis” OR “pre-exposure chemo-prophylaxis” OR “PrEP” OR “chemoprevention” OR “antiretroviral prophylaxis” OR “anti-retroviral prophylaxis” OR “anti retroviral prophylaxis” OR “HIV prophylaxis”) AND (“HIV testing” OR “HIV test” OR “HIVST” OR “HIV screening” OR “HIV screen” OR “HIV diagnos*” OR “HIV serodiagnos*”) AND (“HIV” OR “human immunodeficiency virus” OR “human immuno-deficiency virus” OR “human immunedeficiency virus” OR “human immune deficiency virus”) AND (“Nonprescription Drugs” OR “nonprescription” OR “medication” OR “med” OR “over the counter” OR “over-the-counter” OR “OTC” OR “without a prescription” OR “pharmacist-prescribed” OR “pharmacy” OR “pharmacies” OR “pharmac” OR “pharmacy access” OR “pharmacy initiated” OR “pharmacy-based” OR “community pharmacy services” OR “chemist” OR “drug shop” OR “external pick-up-point” OR “external pick up point” OR “Ex-PuPs” OR “Ex PuPs” OR “decentralized medication delivery” OR “DMD” OR “retail pharmac” OR “community pharmac” ) AND (“sub-Saharan Africa” OR “subsaharan Africa” OR “SSA” OR “Angola” OR “Benin” OR “Botswana” OR “Burkina faso” OR “Burundi” OR “Cameroon” OR “Cape Verde” OR “Central African Republic” OR “Chad” OR “Comoros” OR “DRC” OR “Democratic Republic of Congo” OR “Republic of Congo” OR “Cote divoire” OR “Cote d Ivoire” OR “Equatorial Guinea” OR “Eritrea” OR “Ethiopia” OR “Gabon” OR “Gambia” OR “Ghana” OR “Guinea” OR “Guinea-Bissau” OR “Kenya” OR “Lesotho” OR “Liberia” OR “Madagascar” OR “Malawi” OR “Mali” OR “Mauritania” OR “Mauritius” OR “Mozambique” OR “Nambia” OR “Niger” OR “Nigeria” OR “Rwanda” OR “Sao Tome and Principe” OR “Sao Tome & Principe” OR “Senegal” OR “Seychelles” OR “Sierra Leone” OR “South Africa” OR “South Sudan” OR “Sudan” OR “Swaziland” OR “Tanzania” OR “Togo” OR “Uganda” OR “Zambia” OR “Zimbabwe”) |

| **Characteristic** | **Inclusion** | **Exclusion** |
| --- | --- | --- |
| Population (P) | Enrolled adults living with HIV or without HIV and at risk, all ages; providers delivering HIV services; providers at community pharmacies; stakeholders involved in HIV service delivery | People outside sub-Saharan Africa not at HIV risk or living with HIV; providers delivering HIV services outside sub-Saharan Africa |
| Intervention (I) | Community pharmacy-based HIV testing, ART delivery, or PrEP delivery. | Services that are not related to HIV screening, prevention, or treatment. |
| Comparison (C) | Other models of HIV service delivery outcomes of the community pharmacy setting (e.g., HIV clinic). | N/A |
| Outcome (O) | Implementation outcomes (e.g., acceptability, feasibility); effectiveness outcomes (e.g., HIV incidence) | All other outcomes, including sexual behavior outcomes and the uptake of non-HIV-related services. |
| Study design (S) | Qualitative studies (e.g., in-depth interviews, focus group discussions, key informant interviews), quantitative studies (e.g., cross sectional studies, prospective/retrospective cohorts, randomized trials, discrete choice experiments). | Systematic literature reviews & meta analyses |

**Appendix 3. PICOS criteria for study inclusion in the scoping review**

**Appendix 4. Studies identified on differentiated models of community pharmacy-based HIV service delivery in SSA, by publication year**

**Appendix 5. Traffic light plot - quality assessment of extracted literature using ROBINS-I**


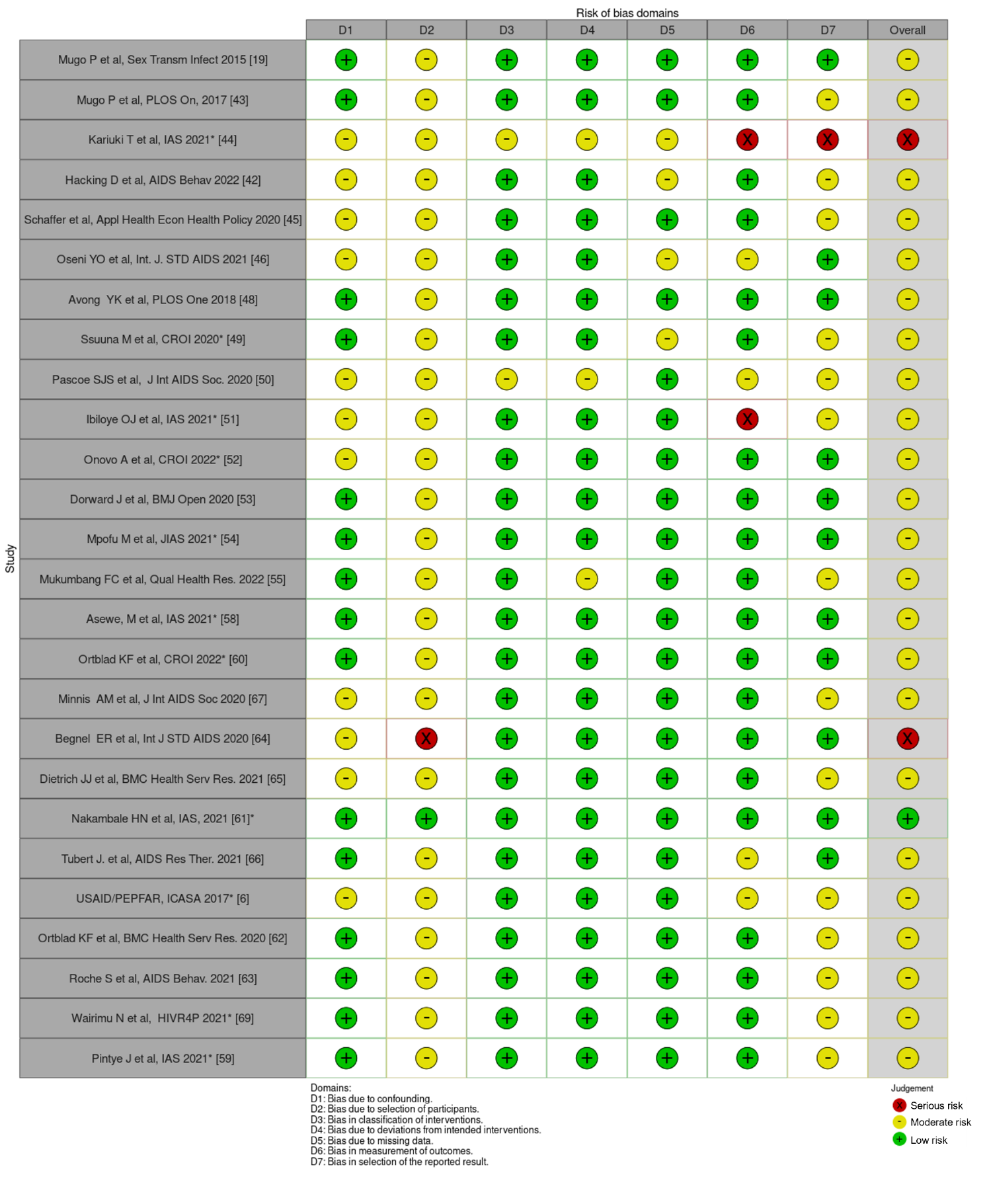


**Appendix 6. Summary plot - quality assessment of extracted literature using ROBINS-I**
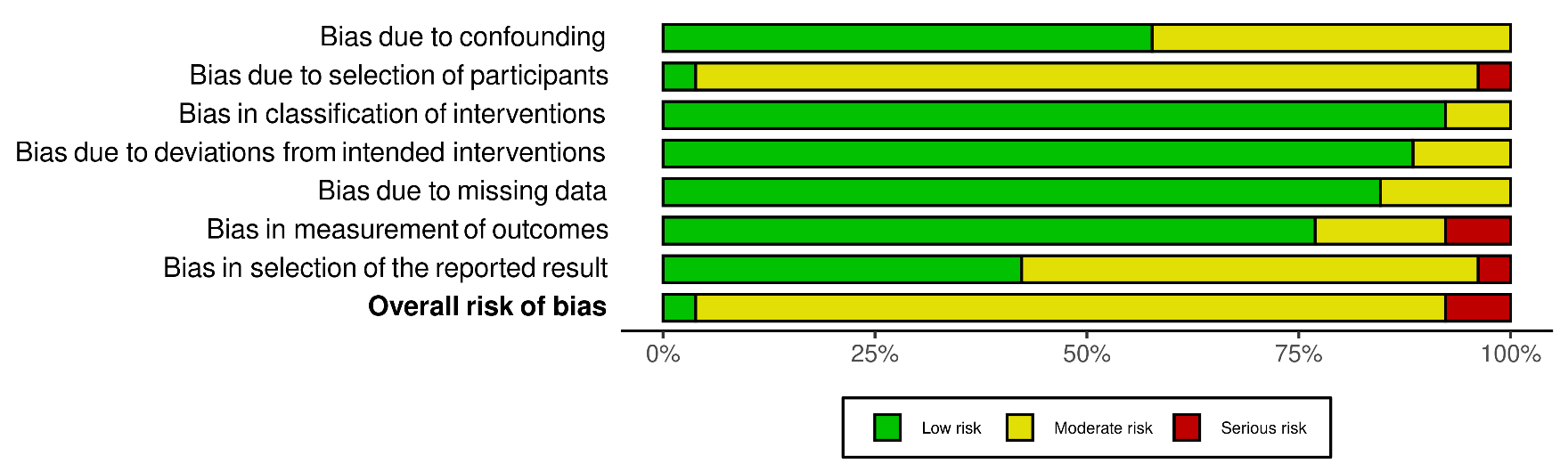


**Appendix 7. Traffic light plot - quality assessment of extracted literature using ROB2
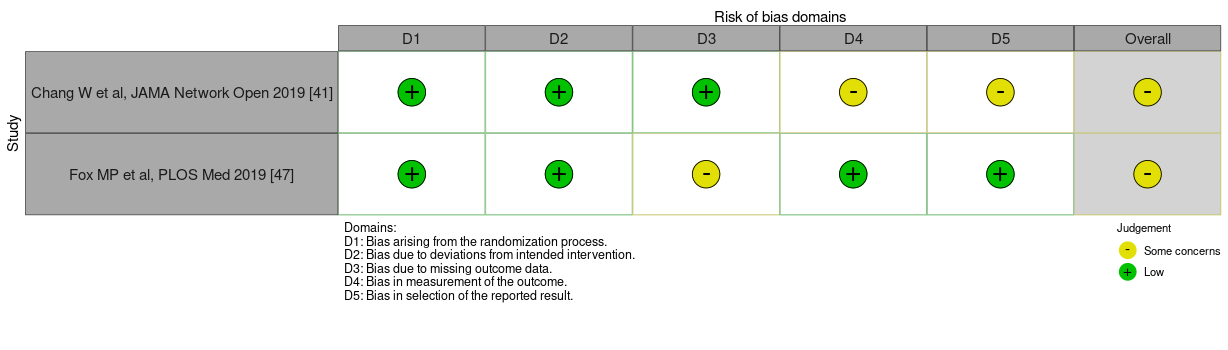
**

**Appendix 8. Summary plot - quality assessment of extracted literature using ROB2**
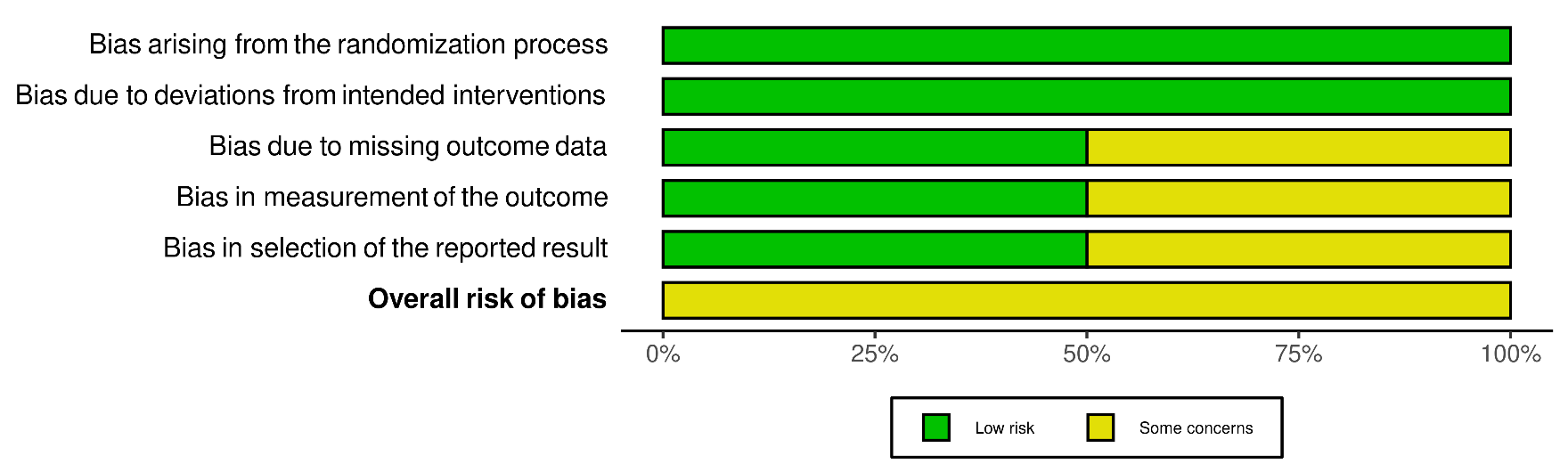

Supplement: Supplementary file 1 — Appendix S1. PRISMA‐ScR Checklist. Appendix S2. Search teams for the scoping review on models of pharmacy‐delivered HIV services in sub‐Saharan Africa. Appendix S3. PICOS criteria for study inclusion in the scoping review. Appendix S4. Studies identified on differentiated models of community pharmacy‐based HIV service delivery in SSA, by publication year. Appendix S5. Traffic light plot ‐ quality assessment of extracted literature using ROBINS‐I. Appendix S6. Summary plot ‐ quality assessment of extracted literature using ROBINS‐I. Appendix S7. Traffic light plot ‐ quality assessment of extracted literature using ROB2. Appendix S8. Summary plot ‐ quality assessment of extracted literature using ROB2. [file JIA2-25-e26027-s001.docx]
